# Scutellarin Alleviates Bone Marrow Mesenchymal Stromal Cellular Senescence via the Ezh2‐Nrf2 Signalling Axis in Diabetes‐Induced Bone Loss

**DOI:** 10.1111/cpr.13790

**Published:** 2024-12-12

**Authors:** Tiantian Wang, Jiehao Chen, Bo Qu, Dong Zhou, Zhen Hong

**Affiliations:** ^1^ Department of Neurology Institute of Neurology and Disease, West China Hospital of Sichuan University Chengdu China; ^2^ Institute of Brain Science and Brain‐Inspired Technology of West China Hospital, Sichuan University Chengdu China; ^3^ Department of Neurology Chengdu Shangjin Nanfu Hospital Chengdu China; ^4^ Animal Laboratory Center, West China Hospital, Sichuan University Chengdu China; ^5^ Department of Orthopedics The First Affiliated Hospital of Chengdu Medical College Chengdu China

**Keywords:** cellular senescence, diabetic osteoporosis, EZH2/H3K27me3, Keap1‐Nrf2, LepR+MSCs, scutellarin

## Abstract

Currently, there is no specific treatment for diabetes‐induced osteoporosis (DOP). Our study identified diabetes‐induced cellular senescence, marked by elevated activity of senescence‐associated β‐galactosidase. Targeting senescent cells holds promise for osteoporosis treatment. We demonstrated that scutellarin (SCU) effectively mitigated bone loss in DOP mice, and co‐treatment with SCU significantly reduced diabetes‐induced senescence in LepR+MSCs. Furthermore, our research highlighted the role of Nrf2 in SCU's anti‐senescence effects on bone. The deletion of Nrf2 impaired SCU's ability to alleviate DOP. Mechanistically, SCU enhances Ezh2 expression and increases H3K27me3 activity at the Keap1 promoter region, leading to Keap1 repression and enhanced Nrf2‐ARE signalling. Additionally, SCU notably inhibited cellular senescence and diabetes‐related osteoporosis, these effects were significantly reduced in Ezh2^LepRcre^ conditional knockout models. These findings suggest that the Ezh2‐Nrf2 signalling axis is crucial for mediating SCU's beneficial effects in this context. Overall, our discoveries provide insights into the mechanisms underlying DOP and propose a potential preventive strategy for this condition.

## Graphical abstract

The Ezh2‐Nrf2 signalling axis plays a crucial role in mediating the beneficial effects of SCU under DOP conditions.

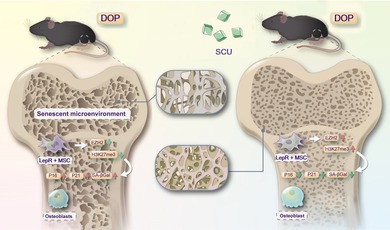

**Abbreviations**
AREs: antioxidant response elements
BMP: bone morphogenetic protein
BMSCs: Bone marrow‐derived mesenchymal stem cells
BV/TV: rabecular bone volume
DOP: diabetes‐induced osteoporosis
H3K27me3: trimethylation of histone 3 at lysine 27
HFD: high‐fat diet
Keap1: Kelch‐like ECH‐associated protein 1
LepR+: leptin receptor‐positive
MSCs: marrow stromal cells
PRC2: polycomb repressive complex 2
ROS: reactive oxygen species
SA‐βgal: Senescence‐associated β‐galactosidase
SCU: scutellarin
STZ: streptozotocin
Tb.N: trabecular number
Tb.Sp: trabecular separation
Tb.Th: trabecular thickness
Trap: tartrate‐resistant acid phosphatase

## 1 Introduction

The prevalence of diabetes, especially type 2 diabetes, rises with age [1]. People with diabetes face a higher risk of developing age‐related issues like frailty, mild cognitive impairment, and osteoporosis (OP). One potential factor contributing to bone defects in diabetic osteoporosis (DOP) patients is the balance between increased bone resorption and decreased bone formation [2, 3]. However, the exact mechanisms driving bone mass reduction in high‐glucose environments are still largely unclear.

Recent studies indicate that cellular senescence significantly contributes to the pathophysiology of OP [4, 5]. Senescent cells display increased lysosomal β‐galactosidase (SA‐βgal) activity and upregulate tumour suppressor genes such as p16 and p15INK4b, leading to cell cycle arrest [6, 7, 8]. Additionally, these cells release factors collectively known as the senescence‐associated secretory phenotype (SASP), which can negatively affect neighbouring cells and disrupt normal tissue function [5, 9, 10, 11]. High‐glucose conditions are known to induce cellular senescence, accelerating this process and decreasing the turnover of bone and blood vessels, thereby exacerbating bone changes in older mice [12, 13, 14]. However, the specific mechanisms through which elevated glucose levels influence bone‐forming cells remain to be fully understood, which is essential for identifying potential therapeutic targets for OP treatment.

Bone marrow‐derived mesenchymal stem cells (BMSCs) serve as precursors for various cell types, including osteoblasts and adipocytes [15]. Current literature indicates that MSCs become senescent under high‐glucose conditions, with an increase in SA‐β‐gal‐positive MSCs during osteogenic differentiation [16, 17]. Furthermore, leptin receptor‐positive (LepR+) cells in the bone marrow are key stem/progenitor cells involved in bone remodelling and are a primary source of both bone and adipocytes [18, 19, 20, 21]. Previous research has shown that glucocorticoids can induce senescence in LepR+ cells, potentially contributing to glucocorticoid‐induced OP [4, 22]. However, the specific effects of high glucose on LepR+ MSCs remain unclear, warranting further investigation.

Nrf2 is a key transcription factor that regulates antioxidant genes, playing a vital role in maintaining cellular redox balance and defending against oxidative stress [23]. Normally, Nrf2 is kept in check by its inhibitor, Keap1, which facilitates its degradation. However, during oxidative stress, the interaction with Keap1 is disrupted, allowing Nrf2 to activate protective responses [24, 25]. This Keap1‐Nrf2 pathway is significant not only for delaying cellular senescence but also for protecting against various diseases, including OP [26, 27, 28, 29, 30, 31]. Recent studies highlight the importance of epigenetic modifications, such as DNA and histone methylation, in regulating the Keap1‐Nrf2 interaction [32]. Specifically, Ezh2, a component of the polycomb repressive complex 2 (PRC2), plays a crucial role in mesenchymal stem cells by trimethylating histone 3 at lysine 27 (H3K27me3), which helps mitigate senescence processes [4, 33]. These insights suggest that targeting the Nrf2 pathway and understanding its epigenetic regulation could provide therapeutic avenues for conditions like OP.

Scutellarin (SCU), a natural flavone derived from Huang Qin, is recognised for its antioxidant and anti‐inflammatory properties [34, 35, 36, 37, 38]. It effectively treats diabetes‐related conditions by reducing inflammation via the Nrf2/HO‐1 signalling pathway and protecting vascular endothelial cells from high glucose‐induced damage through PINK1/Parkin‐mediated mitophagy [39, 40]. Additionally, SCU enhances liver health by reducing hepatocyte apoptosis and has a favourable safety profile with minimal side effects [34, 41]. It also promotes osteoblast proliferation and inhibits osteoclast formation, although its effects on DOP remain unclear [42, 43]. Further research is needed to delve into this potential relationship.

In this study, we explored the therapeutic potential of SCU in alleviating DOP. Our findings showed that SCU treatment effectively influenced the senescence of LepR+ MSCs in the bone marrow of mice. Additionally, our results underscore the crucial role of the Ezh2‐Keap1‐Nrf2 pathway in mediating SCU's effects on senescent LepR+ MSCs. These significant findings strongly indicate that SCU may serve as a promising preventive strategy for DOP.

## 2 Results

### 2.1 SCU Supplementation Prevents Diabetes‐Related Bone Loss in Mice

**FIGURE 1:**
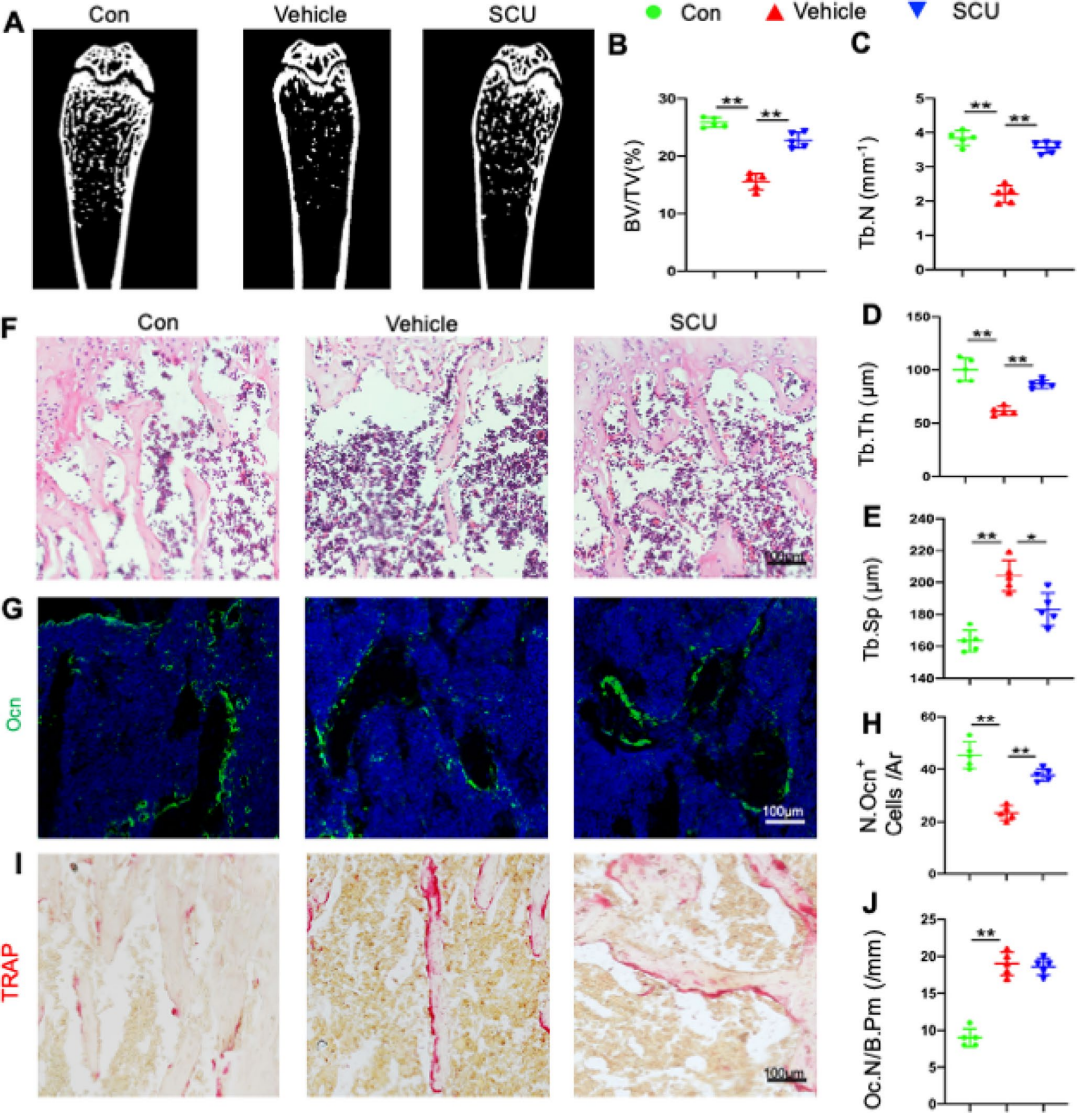
SCU alleviated diabetes‐induced bone mass loss and impaired bone formation. (A) Representative micro‐CT images of distal femurs. (B–E) Quantification of the trabecular bone volume fraction (BV/TV), trabecular bone number (Tb.N), trabecular bone thickness (Tb.Th) and trabecular separation (Tb.Sp). (F) Staining of representative sections of distal femoral growth plates was performed using haematoxylin and eosin. (G) Representative images of immunofluorescence staining of femur sections using antibodies against osteocalcin (Ocn) (green). (H) Quantitative analysis of Ocn^+^ cells in primary trabeculae per mm^2^ tissue area (N.Ocn^+^ cells/Ar). (I) TRAP staining image of femoral sections. (J) Quantified numbers of TRAP^+^ cells (Oc.N/B.Pm/mm). Ocn, osteocalcin; TRAP, tartrate‐resistant acid phosphatase; *p < 0.05, **p < 0.01 by one‐way ANOVA.

SCU has been shown to prevent bone loss in DOP mice, as indicated by increases in trabecular bone volume (BV/TV), trabecular number (Tb.N), and trabecular thickness (Tb.Th), along with a decrease in trabecular separation (Tb.Sp) compared with the vehicle group (Figure 1A–E). No significant differences in cortical bone parameters were noted between control mice and those treated with streptozotocin (STZ) and a high‐fat diet (HFD) (Figure S1A–D). HE staining further confirmed SCU's role in preventing bone loss (Figure 1F), suggesting its potential as a preventive strategy for DOP in wild‐type mice. We also aimed to evaluate the effects of osteoblast and osteoclast activity on bone loss mitigation in SCU‐treated DOP mice. Immunofluorescence analysis of Ocn revealed a notable increase in the number of osteoblasts per bone perimeter (N.Ocn + Cells/Ar) in SCU‐supplemented mice compared with the vehicle group (Figure 1G,H). Furthermore, SCU co‐treatment significantly elevated serum P1NP levels, a marker of bone formation (Figure S1E). Interestingly, no significant changes in bone resorption were observed between the SCU and vehicle groups, as shown by serum CTX‐1 levels (Figure S1F) and TRAP staining (Figure 1I,J). Overall, these findings indicate that SCU mitigates DOP primarily by enhancing bone formation.

### 2.2 SCU Eliminates Senescent Cells Induced by Diabetes

To explore how SCU supplementation alleviates DOP, we analysed cellular senescence and the SASP in the treated mice, as shown in Figure 1. In the bone tissues of SCU‐supplemented mice, we observed significant reductions in markers of cellular senescence and SASP compared with vehicle‐treated mice. Specifically, there were decreased expression levels of p16 and p21 in the bone tissues (Figure S2A,B), alongside reduced mRNA levels of TNF‐α and IL‐6 (Figure S2C,D). These results suggest that SCU may improve DOP by inhibiting cellular senescence and the SASP.

**FIGURE 2:**
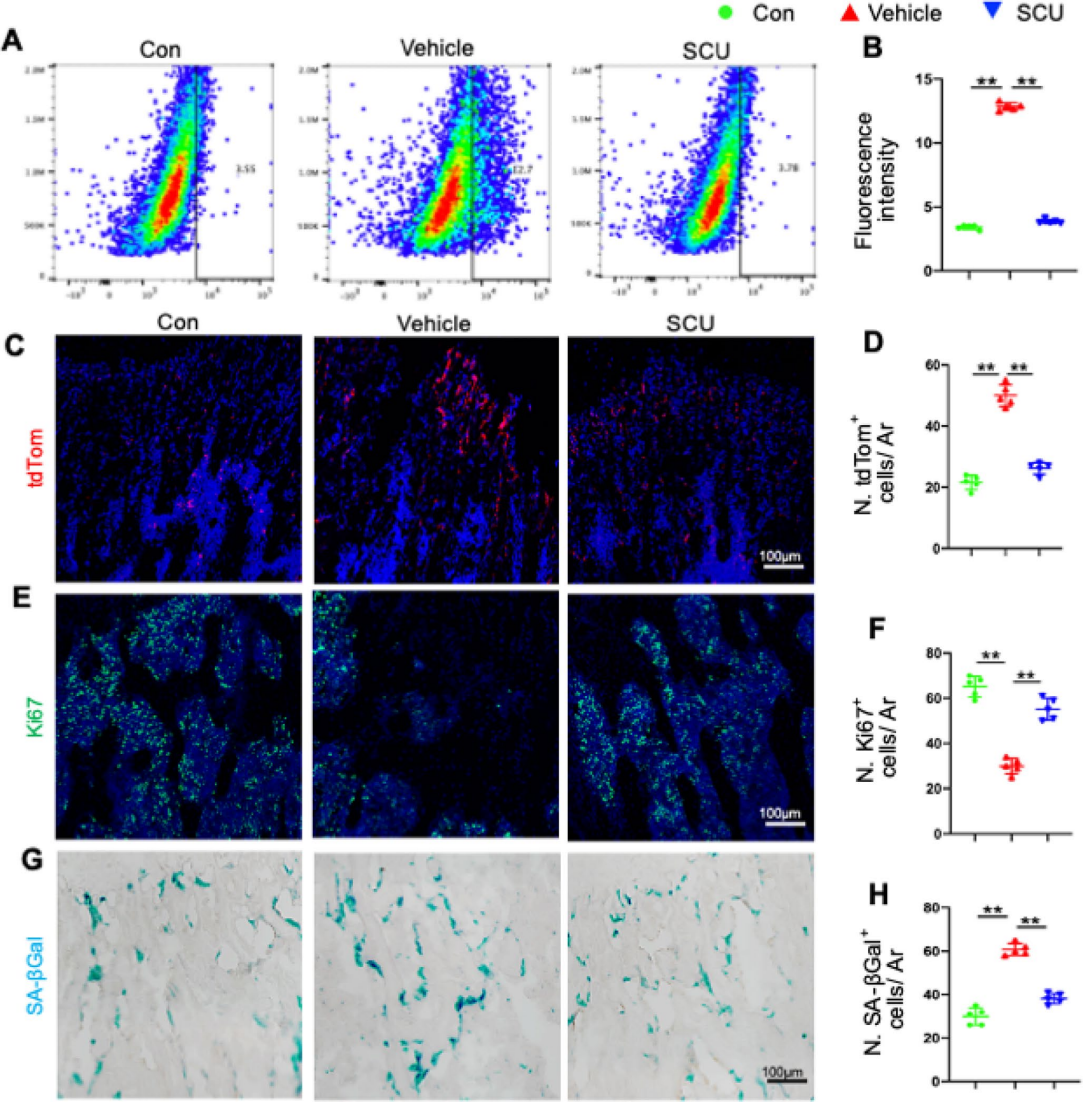
SCU alleviated diabetes‐induced cellular senescence in the bone marrow microenvironment. (A) Representative images of the flow cytometry analysis. (B) Relative fluorescence intensity of the tdtomato+ cells. (C) Representative images of the tdTom‐stained femoral bone sections. (D) Quantification of the percentage of cells that expressed tdTom. (E,F) Representative images of immunofluorescence staining of Ki67^+^ cells and quantified numbers of Ki67^+^ cells (green) in primary trabeculae per mm^2^ tissue area (N. Ki67^+^ cells/Ar). (G) Representative images of SA‐βGal‐stained femoral bone sections. (H) Quantitative analysis of SA‐βGal^+^ cells (blue) in primary trabecular tissue per mm^2^ tissue area (N. SA‐βGal^+^ cells/Ar). Nuclei were stained blue with DAPI. GP, growth plate; Ar, tissue area; tdTom, tdTomato; Ar, tissue area. *p < 0.05, **p < 0.01 by one‐way ANOVA.

Using a murine senescence reporter strain, p16tdTomato, we detected p16‐expressing cells (tdTomato+) at the single‐cell level. Flow cytometry analysis showed a significant increase in tdTomato+ cells in vehicle‐treated mice compared with control mice, whereas the percentage of tdTomato+ cells was notably lower in the SCU group (Figure 2A,B). This indicates that diabetes induces cellular senescence in the metaphysis. Further investigations revealed a significant increase in SA‐βGal+ cells and a decrease in Ki67+ cells in the metaphysis of diabetic mice compared with controls (Figure 2E–H). In contrast, SCU treatment reduced the number of SA‐βGal+ and tdTomato+ cells while increasing Ki67+ cells compared with vehicle treatment (Figure 2C–H).

These findings collectively indicate that SCU has the potential to mitigate bone loss and cellular senescence induced by diabetes.

### 2.3 SCU Prevents Senescence of LepR^+^ MSCs Induced by Diabetes Through Upregulation of Nrf2

**FIGURE 3:**
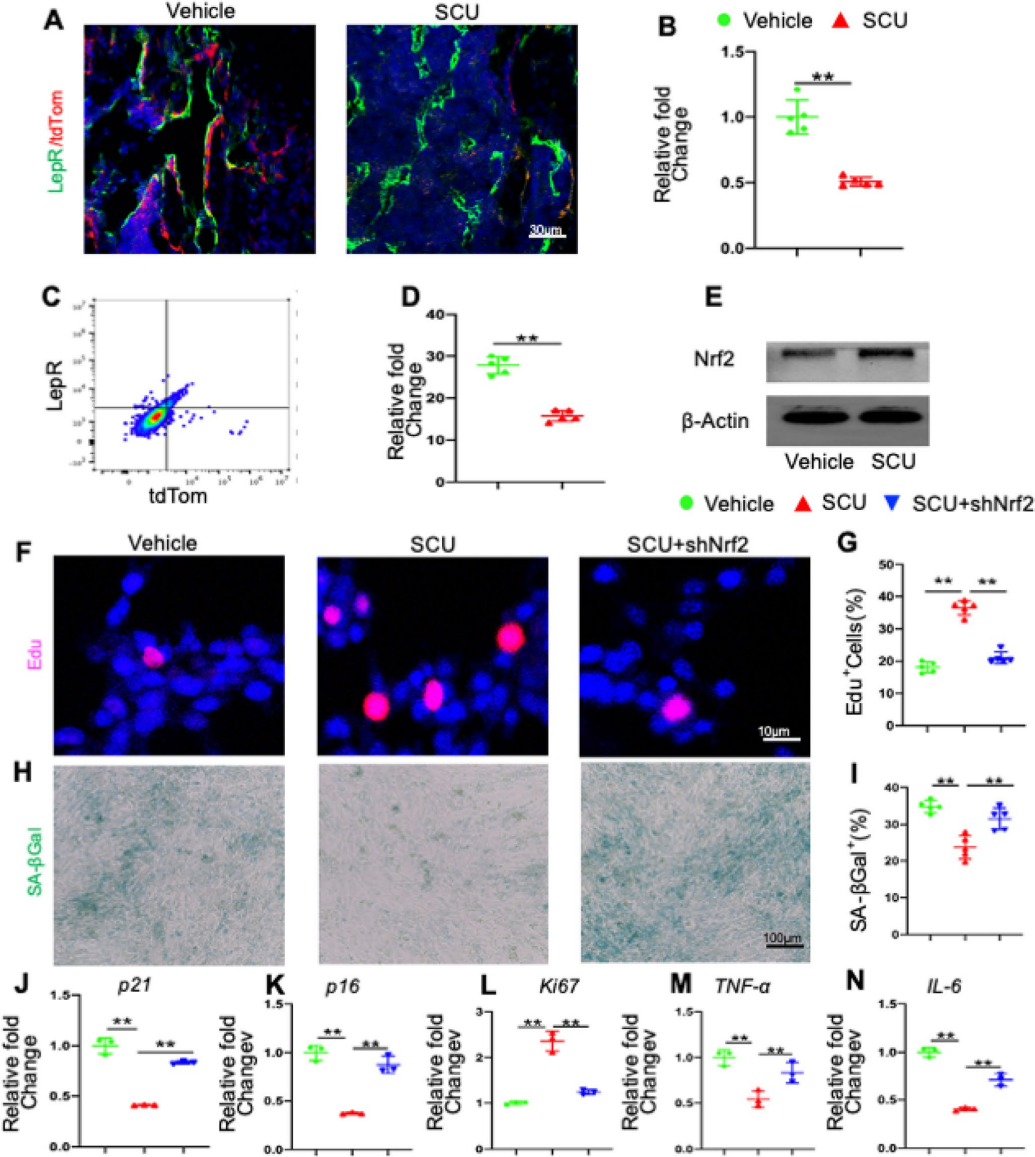
SCU suppresses diabetes‐induced senescence of LepR^+^ MSCs by upregulating Nrf2. *p16* ^*tdTom*^ DOP mice were treated with citrate buffer or SCU daily for 8 weeks. (A) Immunofluorescence staining of femur sections using antibodies against LepR. Red: tdTom^+^ cells; Green: LepR^+^; DAPI stains nuclei blue. (B) Quantitative analysis of the percentage of LepR+tdTom+ cells among all LepR+ primary trabecular cells per mm^2^ tissue area (% tdTom^+^ LepR+ cells/Ar). (C) Representative images of the flow cytometry analysis. (D) The relative fold changes in the number of tdTom and LepR double‐positive cells in the vehicle and SCU groups. (E) Representative immunoblotting images showing the protein expression levels of Nrf2. (F) Representative images of EdU staining. (H) Representative images of in vitro SA‐βGal staining of CD45^−^tdTom^+^ cells. (G and I) Quantification of the percentage of cells that expressed EdU and SA‐βGal relative to the total number of sorted LepR+ CD45‐ cells. (J–N) Representative qRT–PCR analyses of p21, p16, Ki67, TNF‐α and IL‐6 mRNA expression in CD45^−^LepR^+^ cells. Ar, tissue area; tdTom, tdTomato. *p < 0.05, **p < 0.01 by one‐way ANOVA.

LepR serves as a prominent marker for BMSCs, with roughly 0.3% of bone marrow cells expressing it. In adult organisms, LepR+ MSCs play a crucial role in bone remodelling. Utilising p16tdTomato mice enabled us to identify the cell types that underwent senescence and were eliminated by SCU. We conducted co‐staining of bone tissue sections using tdTomato and the MSC‐specific marker LepR. Notably, the combination treatment with SCU led to a reduction in the number of LepR+ MSCs expressing tdTomato (Figure 3A,B) compared with the vehicle group. To further assess the impact of SCU on LepR+ MSC senescence, we positively selected tdTomato and LepR+ MSCs from the bone marrow (Figure 3C). The frequency of tdTomato+ LepR+ cells was lower in SCU‐treated mice than in those treated with the vehicle (Figure 3D). These results indicate that SCU co‐treatment initially mitigated the senescence of LepR+ MSCs. Senescent cells exhibit a reduced capacity to initiate antioxidant signalling in response to stress, which is linked to decreased expression of Nrf2, a key regulator of oxidative stress [44]. Consistently, we observed that LepR+ MSCs in the SCU group expressed higher levels of Nrf2 compared with those in the vehicle‐treated group (Figure 3E). To explore the essential role of Nrf2 in SCU's effects, we directly induced senescence in LepR+ MSCs by exposing them to high glucose levels. Following previous studies, the cells were treated with 25.5 mmol·L^−1^ glucose, a concentration that mimics the diabetic environment in vivo [28]. Moreover, cellular senescence was significantly alleviated in SCU‐supplemented LepR+ MSCs, as shown in Figure 3F,H; however, these changes were not significantly different in SCU‐supplemented shNrf2 cells compared with vehicle‐treated shNrf2 cells (Figure 3F–I). Additionally, qRT–PCR analysis revealed that mRNA levels of senescence markers p21 and p16 were significantly lower in the SCU group than in the vehicle group (Figure 3J,K), while Ki67 mRNA levels were notably higher in the SCU group (Figure 3L). As illustrated in Figure 3M,N, levels of TNF‐α and IL‐6 in LepR+ MSCs from the SCU group were reduced compared with the vehicle group. Silencing Nrf2 inhibited the activation of the Nrf2 signalling pathway induced by SCU, thus blocking its anti‐senescence effects (Figure 3F–N). These findings support the idea that SCU alleviates diabetes‐induced senescence in LepR+ MSCs through the upregulation of Nrf2.

### 2.4 Nrf2 Depletion Largely Blocks the Inhibitory Effects of SCU on DOP

**FIGURE 4:**
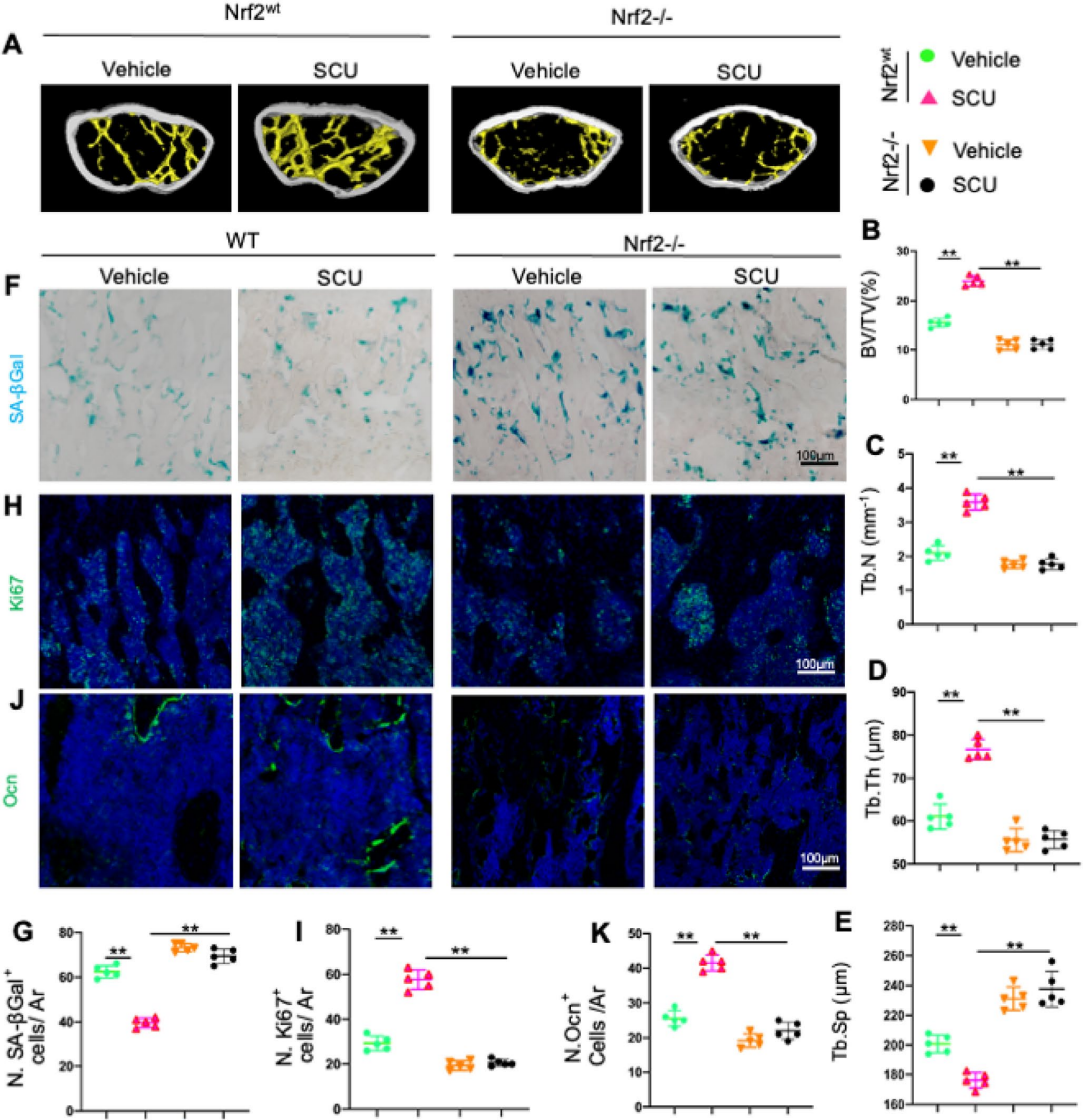
Blocking Nrf2 impairs the SCU‐mediated alleviation of DOP. *Nrf2* ^*wt*^ mice and *Nrf2* ^*−/−*^ mice were divided into vehicle (DOP mice) and SCU (DOP + SCU) groups. (A) Representative micro‐CT images of the distal femurs. (B–E) Quantitative analyses of the trabecular bone volume fraction (BV/TV), the trabecular bone number (Tb.N), the trabecular bone thickness (Tb.Th) and the trabecular separation (Tb.Sp). (F) Representative images of SA‐βGal‐stained femoral bone sections. (G) Quantitative analysis of SA‐βGal^+^ cells (blue) in primary trabecular tissue per mm^2^ tissue area (N. SA‐βGal^+^ cells/Ar). (H and I) Representative images of immunofluorescence staining of Ki67^+^ cells and quantified numbers of Ki67^+^ cells (green) in primary trabeculae per mm^2^ tissue area (N. Ki67^+^ cells/Ar). (J) Representative images of immunofluorescence staining of femur sections using antibodies against osteocalcin (Ocn) (green). (K) Quantitative analysis of Ocn^+^ cells in primary trabeculae per mm^2^ tissue area (N.Ocn^+^ cells/Ar). Ar, tissue area. *p < 0.05, **p < 0.01 by two‐way ANOVA.

SCU‐induced bone formation was completely abolished in Nrf2−/− mice, as demonstrated by Micro‐CT imaging (Figure 4A–E). SA‐βGal and Ki67 staining confirmed that the anti‐senescence effects of SCU were significantly reduced in these Nrf2 knockout models (Figure 4F,H). Additionally, the increase in osteoblasts in the periosteum following SCU treatment was diminished in Nrf2−/− mice (Figure 4J,K). These findings strongly indicate that Nrf2 is crucial for mediating the beneficial effects of SCU on OP related to diabetes.

### 2.5 SCU Upregulates Nrf2 via the Repression of Keap1 by Increasing Ezh2

**FIGURE 5:**
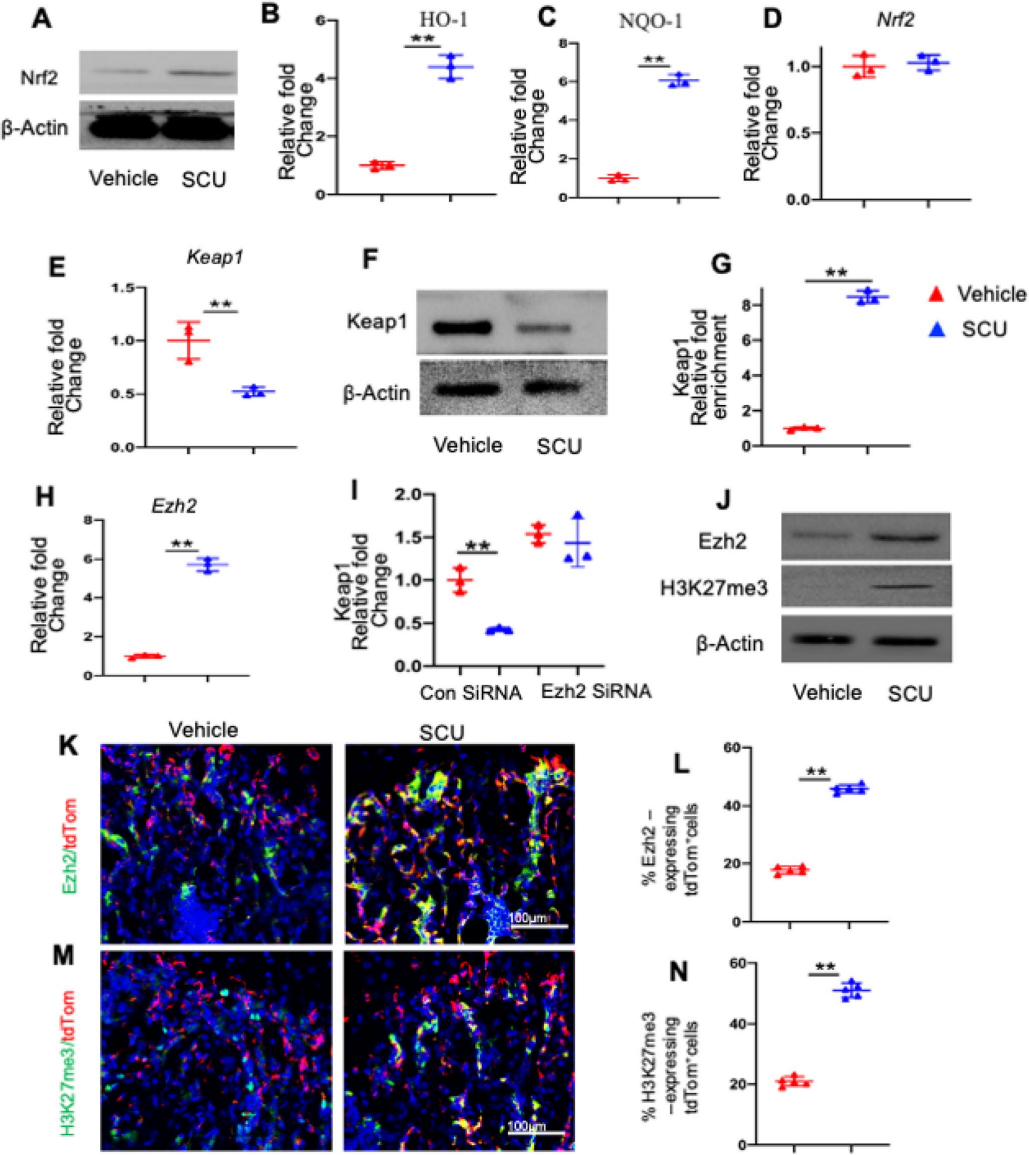
SCU upregulated Nrf2 via the repression of Keap1 by recruiting Ezh2. (A) Representative immunoblotting images showing the protein expression levels of Nrf2. (B‐E) Representative qRT–PCR analyses of HO‐1, NQO‐1, Nrf2, and Keap1 mRNA expression in CD45^−^LepR^+^ cells. (F) Representative immunoblotting images showing the protein expression levels of Keap1. (G) ChIP and input DNA were measured using real‐time PCR with specific primers targeting the promoter regions of the indicated genes. (H) Representative qRT–PCR analyses of Ezh2 mRNA. (I) Relative Keap1 mRNA levels were compared in SCU‐treated LepR+MSCs transfected with EZH2 siRNA or control siRNA. (J) Representative immunoblotting images showing the protein expression levels of Ezh2 and H3K27me3. (K,M) We crossed the *LepR‐Cre* strain with the *Rosa‐LSL‐tdTom* strain. Representative confocal images of immunofluorescence staining of femur sections. Red: TdTom^+^ cells; Green: Ezh2^+^ or H3K27me3^+^ cells; DAPI stains nuclei blue. (L,N) Quantification of the percentage of tdTom‐expressing Ezh2^+^ cells and H3K27me3^+^ cells to all tdTom+ cells. tdTom, tdtomato. *p < 0.05, **p < 0.01.

To explore the mechanism by which SCU upregulates Nrf2, LepR+MSCs isolated from DOP mice were treated with varying concentrations of 50 μM SCU for 48 h. The expression levels of Nrf2 were assessed using Western blotting and real‐time PCR. Our research demonstrated that SCU enhances the transcriptional activity of Nrf2 (Figure 5A) and increases the expression of its downstream genes, such as HO‐1 and NQO‐1 (Figure 5B,C). These findings indicate that SCU enhances the antioxidant capacity of LepR+MSCs by activating the Nrf2‐ARE pathway.

Surprisingly, SCU administration did not significantly alter Nrf2 mRNA levels, as shown in Figure 5D, suggesting that SCU may regulate Nrf2 at the posttranslational level. Keap1, the primary regulator of Nrf2 at this level, was then analysed via Western blotting and real‐time PCR. The study revealed a notable decrease in Keap1 protein expression following SCU treatment (Figure 5F), accompanied by a significant reduction in Keap1 mRNA levels (Figure 5E). Given the regulation of intracellular Keap1 through DNA methylation and histone modification, we hypothesized that SCU might suppress Keap1 transcription by inducing hypermethylation of DNA or histones in the Keap1 promoter complex. However, DNA methylation analysis of the Keap1 promoter following SCU treatment showed no significant changes (data not shown), suggesting that the mechanism by which SCU suppresses Keap1 expression does not involve alterations in DNA methylation.

We further assessed H3K27me3 levels in the Keap1 promoter region of SCU‐treated LepR+MSCs using a ChIP‐qPCR assay. The results indicated a significant increase in H3K27me3 levels in the Keap1 promoter region of SCU‐treated LepR+MSCs compared with the vehicle‐treated controls (Figure 5G). Next, we explored whether SCU increases Ezh2 levels to suppress Keap1 expression. Ezh2 mRNA levels were indeed higher in the SCU‐treated group than in the vehicle group (Figure 5H). However, the knockdown of Ezh2 significantly attenuated the effects of SCU on Keap1 mRNA (Figure 5I). Additionally, we observed a significant increase in Ezh2 protein levels, along with elevated H3K27me3 protein levels, in LepR+MSCs compared with the vehicle group (Figure 5J). These results indicate that SCU can inhibit Keap1 transcription through increased Ezh2 expression.

To further validate these findings in vivo, we utilised a LepRcre‐tdTomato mouse diabetes model, created by crossing the LepR‐Cre strain with the Rosa‐LSL‐tdTomato strain. Immunofluorescence staining of Ezh2 and H3K27me3 revealed a significant increase in the percentages of tdTomato+Ezh2+ and tdTomato+H3K27me3+ cells after SCU treatment compared with the vehicle group (Figure 5K–N). These findings suggest that Ezh2/H3K27me3 play a crucial role in alleviating the senescence of LepR+MSCs induced by SCU.

### 2.6 Deletion of *Ezh2* in LepR^+^ MSCs Blocks the Ability of the SCU to Alleviate Senescence

**FIGURE 6:**
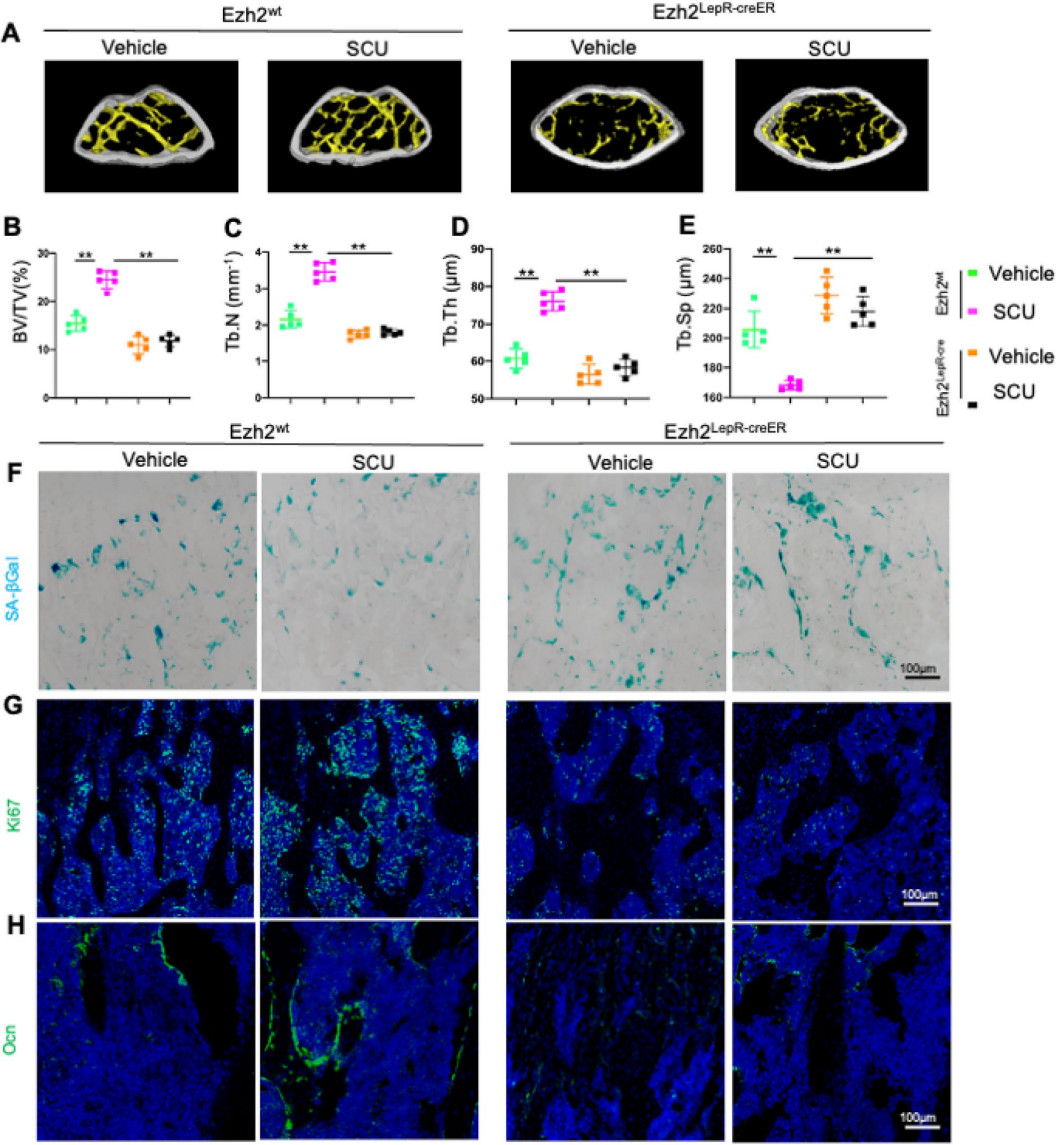
Deletion of *Ezh2* in LepR^+^ MSCs impaired the ability of the SCU to alleviate senescence. *Ezh2* ^*wt*^ mice and *Ezh2* ^*LepR‐cre*^ DOP mice were divided into vehicle and SCU groups. (A) Representative micro‐CT images of the distal femurs. (B–E) Quantitative analyses of the trabecular bone volume fraction (BV/TV), the trabecular bone number (Tb.N), the trabecular bone thickness (Tb.Th) and the trabecular separation (Tb.Sp). (F) Representative images of SA‐βGal‐stained femoral bone sections. (G) Representative images of immunofluorescence staining and quantified numbers of Ki67^+^ cells (green). (H) Representative images of immunofluorescence staining of femur sections using antibodies against osteocalcin (Ocn) (green). *p < 0.05, **p < 0.01 by two‐way ANOVA.

To further explore the role of Ezh2/H3K27me3 in SCU's anti‐senescence effects in vivo, we generated Ezh2^LepRcre^ mice, deleting Ezh2 specifically in LepR+ MSCs. Our findings showed that SCU significantly increased BV/TV and Tb.N in Ezh2^wt^ DOP mice, but not in Ezh2 KO mice (Figure 6A–C), indicating a critical role for Ezh2. This pattern was also evident in Tb.Th (Figure 6D) and trabecular separation, which SCU could reverse in Ezh2^wt^ but not Ezh2 KO mice (Figure 6E). Furthermore, SA‐βGal staining highlighted that Ezh2 deletion obstructed SCU's senescence‐alleviating effects, with Ezh2 KO mice showing increased SA‐βGal+ cells and decreased Ki67+ cells compared with Ezh2wt counterparts (Figures 6F,G and S3A,B). Additionally, the number of osteocalcin‐positive (Ocn+) cells increased in Ezh2^wt^ SCU‐treated mice but remained unchanged in Ezh2 KO mice (Figures 6H and S3C). These results confirm that Ezh2 deletion in LepR+ MSCs diminishes the anti‐senescence effects of SCU.

## 3 Discussion

In our study, we discovered that SCU treatment significantly improved femur trabecular parameters and enhanced osteogenic differentiation and bone formation in DOP. Consistent with other DOP models, our micro‐CT analysis showed no changes in cortical parameters compared with the Con group, possibly due to the short modelling period not being sufficient for cortical remodelling [28, 45]. SCU effectively alleviated cellular senescence in the bone metaphysis by targeting and eliminating senescent LepR+ MSCs, thereby inhibiting bone loss. Mechanistically, SCU promotes Nrf2 accumulation and activates its transcriptional targets by increasing Ezh2 and enhancing H3K27me3 at the Keap1 promoter, leading to Keap1 repression and reduced Nrf2 degradation. These findings illuminate the susceptibility of adult LepR+ MSCs to diabetes, contributing to senescent cell accumulation and offering insights into diabetes's detrimental effects on skeletal development. SCU may serve as a promising preventive strategy for DOP in aging individuals.

Recent studies highlight the dual role of cellular senescence in epithelial regeneration, indicating its complexity [46, 47, 48]. Our research supports the significant role of cellular senescence in bone loss related to DOP, particularly in the context of type 2 diabetes mellitus. We observed elevated SA‐βgal and reduced Ki67+ markers in DOP mice, suggesting high glucose exposure induces senescence and inhibits proliferation. Additionally, using p16tdTomato reporter mice, we noted a marked decrease in tdtomato+ (p16‐expressing) cells in the metaphysis after 8 weeks of SCU treatment compared with the vehicle group, confirmed by flow cytometry and immunofluorescence. These findings underscore the negative effects of high glucose on bone development in adult mice and suggest that SCU may have therapeutic potential for this condition.

High glucose induces senescence in various cell types, including LepR+ MSCs, which are crucial for bone remodelling and are implicated in conditions like OP [21, 49, 50]. Our flow cytometry analysis showed that LepR+ MSCs exhibited a senescent phenotype, marked by elevated p16 and p21 expression. These senescent cells release cytokines that can worsen stem cell dysfunction. We found that LepR+ MSCs secreted higher levels of SASP components, such as TNF‐α and IL‐6. Importantly, pharmacologically eliminating these senescent cells improved bone mass and quality, suggesting that targeting them could be a therapeutic strategy. Our results indicate that SCU significantly reduced senescent cells in the bone marrow, effectively suppressing the senescence of LepR+ MSCs and their SASP, highlighting SCU's potential in enhancing bone formation therapies and improving the bone marrow microenvironment.

A prior study indicated that SCU increased the activity of the antioxidant enzyme superoxide dismutase while reducing ROS production, highlighting its potential as an antioxidant [51]. Our research revealed that SCU can counteract DOP in mice by inhibiting cellular senescence, partly through the activation of Nrf2. The Nrf2‐ARE signalling pathway is vital for protecting against oxidative stress from various stimuli. Additionally, Nrf2‐deficient mice displayed significant bone loss associated with severe diabetes‐related OP. These findings suggest that Nrf2 is essential in preventing the decline in MSC activity due to senescence and in supporting bone health. Notably, the beneficial effects of SCU on combating senescence‐related bone issues were diminished in Nrf2 knockout models, indicating that SCU's ability to alleviate DOP and senescent cells relies on Nrf2 presence. Our results demonstrate that SCU mitigates the senescence of LepR+ MSCs induced by diabetes by upregulating Nrf2 at the posttranslational level. Keap1 functions as the primary regulator of Nrf2 in this context, and we concentrated on the Keap1–Nrf2 interaction as a key regulatory pathway, resulting in reduced nuclear translocation [24, 25]. Gaining a deeper understanding of these intricate interactions and regulatory mechanisms is crucial for elucidating the broader implications of Nrf2 signalling on cellular functions, particularly concerning oxidative stress, senescence, and bone metabolism. As illustrated in Figure 5, decreased Keap1 levels correlated with enhanced Nrf2 protein stability and increased nuclear translocation. Keap1 regulation primarily involves epigenetic silencing, such as promoter hypermethylation [52]. Moreover, DNA hypermethylation analysis revealed no changes in the Keap1 promoter in the presence of SCU. However, we found that SCU promotes the recruitment of the corepressor Ezh2 to the Keap1 promoter, facilitating H3K27me3 activity in that region. This transcriptional repression of Keap1 leads to reduced Nrf2 degradation. Additionally, several studies have underscored the critical role of Ezh2 in MSC senescence and bone remodelling [4, 53]. The Ezh2‐mediated H3K27me3 epigenetic regulation of MSCs is a well‐documented mechanism in bone metabolism [4, 53]. Our research demonstrates that Ezh2‐H3K27me3 serves as a key epigenetic regulator modulating the inhibition of senescence in LepR+ MSCs during SCU treatment. Ezh2‐H3K27me3 orchestrates the senescence process of LepR+ MSCs in a spatiotemporal manner, aligning with previous findings [4, 53, 54]. The deletion of Ezh2 in LepR+ cells not only led to an increased number of senescent cells in the metaphysis and a reduction in osteogenesis [54] but also negated the anti‐senescent effects of SCU in DOP mice. Our research highlights the significance of the Ezh2–Nrf2 pathway as a crucial regulator mediating how SCU alleviates senescence in LepR+ MSCs.

The results of this research indicate that SCU enhances Ezh2 levels and facilitates H3K27me3 activity at the Keap1 promoter, leading to Keap1 repression and improved Nrf2‐ARE signalling. This activation of Nrf2 by SCU helps eliminate senescent LepR+ MSCs, thereby preventing DOP. Given that SCU is widely recognised as a safe supplement for various health conditions, it presents a promising option for addressing age‐related issues like DOP. Further studies in human subjects will be essential to explore the potential benefits of SCU in this area.

## 4 Limitation of the Study

This study has two key limitations. First, it primarily focuses on SCU's role in regulating oxidative stress and the Keap1–Nrf2 pathway in DOP. Future research should investigate SCU's effects on other organs through specific targets and consider developing drug delivery systems for precise bone targeting. Second, while the study underscores SCU's potential therapeutic benefits, the long‐term safety and possible side effects of extended SCU use have not been thoroughly evaluated. Given Ezh2s influence on a wide range of gene expressions, prolonged modulation could result in unforeseen biological effects. This limitation should be acknowledged and explored in future studies.

## 5 Materials and Methods

### 5.1 Animals and Drug Treatment

We procured 4‐week‐old C57BL/6J mice from Beijing HFK Bioscience Co. Ltd., in Beijing, China and p16tdtomato mice from the Shanghai Model Organisms Center Inc., in Shanghai, China. Nrf2+/− mice, LepR*‐Cre* mice, *Ezh2* ^*flox/flox*^ mice, and *Rosa‐LSL‐tdTomato* mice were obtained from Cyagen Biosciences (Guangzhou, China). LepR*‐Cre* mice were crossed with *Rosa‐LSL‐tdTomato* mice to generate LepR*‐Cre‐tdTomato* mice. *LepR‐Cre* mice were crossed with *Ezh2* ^*flox/flox*^ mice. The offspring were backcrossed with *Ezh2* ^*flox/flox*^ mice to generate LepR*‐Cre::Ezh2* ^*flox/flox*^ mice (*Ezh* ^*LepRcre*^) and *Ezh2* ^*flox/flox*^ (*Ezh2* ^*wt*^) mice. The genotypes of the mice were determined through PCR analysis of genomic DNA extracted from tailor toe biopsies using the following primers: LepR‐Cre forward, 5′‐GTCATGAACTATATCCGTAACCTGG‐3′ and reverse, 5′‐GACAGGCTCTACTGGAATGGAAC‐3′; loxP Ezh2 allele forward, 5′‐GGATAAGAATTACCATGGCAGCC‐3′and reverse, 5′‐TAGCACCGTTTAGAAGGTTTAGC‐3′; Rosa‐LSL‐tdtomato mice forward, 5′‐GGCATTAAAGCAGCGTATCC‐3′and reverse, 5′‐CTGTTCCTGTACGGCATGG‐3′. The p16tdTomato allele was forward (5′‐ATAGGGCTTCTTTCTTGGGTCC‐3′) and reverse (5′‐TCACGTTCATTATAAATGTCGTTCG‐3′). A mouse model of type 2 diabetes was created following the methodology outlined in a previously published study [28, 45]. We developed a mouse model of DOP using a HFD (60% kcal from fat) combined with low‐dose STZ injections. The control group received a regular diet with 10% kcal from fat. After 4 weeks, all groups except the control received intraperitoneal STZ injections (35 mg/kg) for seven consecutive days to induce diabetes. To assess whether SCU supplementation could prevent DOP, the SCU group received intraperitoneal injections of SCU (10 mg/kg) twice a week for 8 weeks, while the vehicle group was treated with citrate buffer. The mice were euthanized for blood and bone analysis. After the skin was removed, the femurs were collected and fixed in 4% paraformaldehyde for further experiments.

The animal experiments were conducted in a specific pathogen‐free facility. The mice were kept in a temperature‐controlled room with a 12:12 h light–dark cycle and provided unrestricted access to food and water. All protocols were carefully reviewed and approved by the Institutional Animal Care and Use Committee of Sichuan University. Each experiment was performed in triplicate and repeated at least three times.

### 5.2 Micro‐CT Analysis

After the animals were sacrificed, the femora were fixed in 4% paraformaldehyde at 4°C. Microcomputed tomography (micro‐CT) analysis was subsequently performed using an NMC‐100 scanner from PINGSHENG (Shanghai, China). The scanning parameters were set at 90 kV and 0.06 mA, with a resolution of 0.015 × 0.015 × 0.017 mm. To determine the trabecular bone parameters in the metaphysis, recon image reconstruction software and Avatar data analysis software were utilised. Trabecular measurements were conducted by consistently obtaining 100 micro‐CT images with an 8‐μm layer spacing.

For three‐dimensional histomorphometric analysis of the spongiosa, cross‐sectional images of the distal femurs were obtained. Regions of interest were defined from 1 to 1.5 mm below the growth plate. The following parameters were analysed: bone volume fraction (BV/TV), Tb.N, Tb.Th, and Tb.Sp. Cortical morphometry analysis was performed on a section at the mid‐diaphysis of the femur to determine cortical parameters.

### 5.3 Immunohistochemistry and Immunofluorescence

After the right femurs were resected, they were fixed in 4% paraformaldehyde overnight and then decalcified using a 20% EDTA decalcification solution. The decalcification solution was renewed every 3–4 days until the bones were completely softened. Following decalcification, the bones were soaked in 20% polyvinylpyrrolidone and sucrose for 24 h to prepare them for immunofluorescence staining. Subsequently, the tissues were embedded in optimal cutting temperature (OCT) compound. For senescence‐associated beta‐galactosidase (SA‐βgal) staining, a β‐Gal staining kit (Cell Biolabs, CBA‐230) was used. For immunofluorescence analysis, the femurs were sectioned into 40‐μm‐thick coronal‐oriented sections and then incubated with primary antibodies overnight at 4°C. The primary antibodies used included Ki67, Ocn, Ezh2, H3K27me3, and LepR, each at varying dilutions. Subsequently, the sections were treated with FITC‐ or TRITC‐conjugated secondary antibodies, and the nuclei were counterstained with DAPI (Sigma). The stained sections were visualised using Nikon (N‐STORM and A1) confocal microscopes. Image analysis was carried out using ImageJ software to quantitatively analyse the fluorescence signals and cellular markers in the bone tissue sections.

### 5.4 Bone Histological Analysis

The femurs were fixed in 4% paraformaldehyde for 24–48 h and then transferred to 20% EDTA at 37°C for decalcification. The decalcification solution was replaced every 3–4 days until the femurs became completely softened. After decalcification, the bones were dehydrated and embedded in paraffin. Approximately 5‐μm‐thick sections were prepared in the sagittal plane. For TRAP staining, the sections were stained with TRAP (Sigma‐Aldrich, St. Louis, MO) and haematoxylin–eosin (H&E). TRAP‐positive multinucleated cells were visualised using an upright metallurgical microscope (Ni‐E, Nikon, Tokyo, Japan). Quantitative histomorphometric analysis of TRAP‐positive osteoclasts was performed using ImageJ software. Furthermore, H&E staining was conducted on the femur sections following the standard protocol. This staining method allows visualisation of the general morphology and histological features of the tissue.

To measure serum CTX‐1, P1NP levels, blood serum was collected via cardiac puncture immediately prior to sacrifice and stored at −80°C. Both were quantified using an enzyme immunoassay (EIA) kit according to the manufacturer's protocol.

### 5.5 Cell Sorting and Flow Cytometry Analysis of LepR+ Cells

Mice were sacrificed to obtain bone marrow cells from their femurs and tibias. The bone was digested using a solution containing collagenase A (2 mg/mL) and trypsin (2.5 mg/mL) in PBS for 30 min. After this initial digestion, the bones were cut into small pieces and transferred to a protease solution for further digestion for 1 h. The resulting supernatant containing the cells was collected for flow cytometry analysis. To remove red blood cells, ACK lysis buffer (Gibco, A1049201) was used, after which the number of cells was quantitatively determined. Flow cytometry analysis was performed using a 6‐laser BD FACS and FACS Diva (BD Biosciences, San Jose, CA, USA). Following the sorting process, the cells were exposed to 25.5 mmol·L^−1^ glucose for 48 h, which was consistent with the diabetic environment in vivo. The direct effects of SCU on LepR+ cells were assessed by subjecting the sorted LepR+ cells with or without the addition of SCU. This method allowed the investigation of the impact of SCU on LepR+ cells and their responses to senescence induction.

### 5.6 RNA Isolation and qRT‐PCR

Total RNA was isolated from the sorted cells and bones using TRIzol reagent (Invitrogen, California, USA). The RNA quality was assessed by measuring the A260/A280 ratio. A PrimeScript RT reagent kit (Takara Bio, Japan) was used for cDNA synthesis. Subsequently, qRT–PCR was conducted with SYBR Premix Ex Taq II kits (Takara Bio, Japan) on a CFX Real‐Time PCR Detection System (Bio‐Rad, California, USA). The mRNA expression levels were normalised to those of GAPDH and analysed using the 2‐ΔΔCT method as previously described [55]. Primers used for qRT‐PCR were as follows: *Ezh2* (5′‐AATCAGAGTACATGCGACTGAGA‐3′) and (5′‐GCTGTATCCTTCGCTGTTTCC3′); *Jmjd3* (5′‐CAACTCCATCTGGCTGTTACTG‐3′) and (5′‐CCTTCTGCAACCAATTCCAG‐3′); *p16* (5′‐CGTACCCCGATTCAGGTGAT‐3′) and (5′‐TTGAGCAGAAGAGCTGCTACGT‐3′); *p21* (5′‐GTGGGTCTGACTCCAGCCC‐3′) and (5′‐CCTTCTCGTGAGACGCTTAC‐3′); *Ki67* (5′‐CTGCCTGCGAAGAGAGCATC‐3′) and (5′‐AGCTCCACTTCGCCTTTTGG‐3′); *GAPDH* (5′‐CTGCTTCACCACCTTCTTGA‐3′) and (5′‐AAGGTCATCCCAGAGCTGAA‐3′). *TNF‐α* (GGGTGTTCATCCATTCTC) and (GGAAAGCCCATTTGAG); *IL‐6* (GAAACCGCTATGAAGTTCCTCTCTG) and (TGTTGGGAGTGGTATCCTCTGTGA); Nrf2 (TCTTGGAGTAAGTCGAGAAGTGT) and (GTTGAAACTGAGCGAAAAAGGC).

### 5.7 ChIP‐qPCR

ChIP‐qPCR was performed using SYBR Green PCR Master Mix and 7900 HT Fast Real‐Time PCR System (Applied Biosystems Corp., Foster City, CA, USA). Primers for Keap1 were used (Keap1‐S1: ATCTGGTAGGTAGGTGCTAT and CTATCAAGAGGCGTGTGG; Keap1‐S2: CCAGCAACGAAATCGGGAGA and GGGAAAGGAGCGGCGATTC). Absolute quantification was performed and enrichment expressed as a fraction of the whole‐cell extract control.

### 5.8 Western Blot Analysis

Cells were lysed using RIPA lysis buffer to extract total protein. The lysate was then centrifuged at 12,000 × g for 20 min at 4°C, and the resulting supernatant was collected for protein concentration determination using the BCA method. For further analysis, 20 μg of protein was loaded onto a polyacrylamide gel (10%) and subjected to electrophoresis. The separated proteins were then transferred onto PVDF membranes through a blotting process. To prevent non‐specific binding, the membranes were blocked with 5% fat‐free milk and incubated overnight at 4°C with primary antibodies. Afterward, secondary antibodies were applied and incubated for 1 h. Bands were visualised using electrochemiluminescence detection reagents. The primary antibodies used in this study were against Ezh2 (Cell Signalling 5246, 1:1000), H3K27me3 (Cell Signalling 9733, 1:1000), and β‐Actin (Beyotime, AF2811).

### 5.9 Statistical Analysis

The data are presented as the means with standard deviations. Multiple comparisons were performed using either one‐way or two‐way analysis of variance (ANOVA) with a post hoc Tukey test. In cases where equal variances were not assumed, Welch's ANOVA with a post hoc Games–Howell test was conducted for multiple comparisons. Statistical analysis was carried out utilising SPSS 26.0 software (SPSS, Chicago, IL, USA). Differences were considered significant at a threshold of *p* < 0.05.

## Author Contributions

Conceptualization: T.W., Z.H., and D.Z. Methodology: T.W. Investigation: T.W. and J.C. Software: J.C. Visualisation: T.W., and J.C. Supervision: T.W. and Z.H. Writing – original draft: T.W. Writing – review and editing: T.W., Z.H., D.Z., and B.Q.

## Ethics Statement

I certify that this article is original, has not been published and will not be submitted elsewhere for publication while being considered by Cell proliferation. The study is not split into several parts to increase the quantity of submissions and is submitted to various journals or to one journal over time. No data have been fabricated or manipulated (including images) to support our conclusions. No data, text, or theories by others are presented as if they were our own.

## Conflicts of Interest

The authors declare no conflicts of interest.

## Supporting information


**Figure S1.** SCU alleviated diabetes‐induced bone mass loss and senescence of cells. (A) Representative micro‐CT images of cortical bone. (B) Cortical bone area (Ct.ar) and cortical bone thickness (Ct.Th) (C), and cortical bone area/tissue area (Ct.ar/Tt.ar) (D). (E,F) The concentrations of P1NP and CTX‐1 were determined in blood samples. *p < 0.05, **p < 0.01 by one‐way ANOVA.
**Figure S2.** SCU alleviated senescence and SASP in bone tissue. Representative qRT–PCR analyses of p21, p16, TNF‐α, and IL‐6 mRNA expression in bone tissue from the Con, Vehicle, and SCU groups (A–D). *p < 0.05, **p < 0.01 by one‐way ANOVA.
**Figure S3.** Blocking *Ezh2* in LepR^+^ MSCs impaired the ability of SCU to alleviate senescence. (A) Quantitative analysis of SA‐βGal^+^ cells (blue) in primary trabecular tissue per mm^2^ tissue area (N. SA‐βGal^+^ cells/Ar). (B) Quantitative analysis of Ki67^+^ cells (green) in primary trabeculae per mm^2^ tissue area (N. Ki67^+^ cells/Ar). (C) Quantitative analysis of Ocn^+^ cells in primary trabeculae per mm^2^ tissue area (N.Ocn^+^ cells/Ar). Ar, tissue area. *p < 0.05, **p < 0.01 by two‐way ANOVA.

## Data Availability

Additional data collected during this study are available from the corresponding author upon reasonable request.
