# Supplementary material for: Scutellarin Alleviates Bone Marrow Mesenchymal Stromal Cellular Senescence via the Ezh2‐Nrf2 Signalling Axis in Diabetes‐Induced Bone Loss
Source: Cell Prolif. 2024 Dec 12;58(4):e13790. doi: 10.1111/cpr.13790 (PMC11969241; doi:10.1111/cpr.13790)
Supplement: Supplementary file 1 — Figure S1. SCU alleviated diabetes‐induced bone mass loss and senescence of cells. (A) Representative micro‐CT images of cortical bone. (B) Cortical bone area (Ct.ar) and cortical bone thickness (Ct.Th) (C), and cortical bone area/tissue area (Ct.ar/Tt.ar) (D). (E,F) The concentrations of P1NP and CTX‐1 were determined in blood samples. *p < 0.05, **p < 0.01 by one‐way ANOVA. Figure S2. SCU alleviated senescence and SASP in bone tissue. Representative qRT–PCR analyses of p21, p16, TNF‐α, and IL‐6 mRNA expression in bone tissue from the Con, Vehicle, and SCU groups (A–D). *p < 0.05, **p < 0.01 by one‐way ANOVA. Figure S3. Blocking Ezh2 in LepR+ MSCs impaired the ability of SCU to alleviate senescence. (A) Quantitative analysis of SA‐βGal+ cells (blue) in primary trabecular tissue per mm2 tissue area (N. SA‐βGal+ cells/Ar). (B) Quantitative analysis of Ki67+ cells (green) in primary trabeculae per mm2 tissue area (N. Ki67+ cells/Ar). (C) Quantitative analysis of Ocn+ cells in primary trabeculae per mm2 tissue area (N.Ocn+ cells/Ar). Ar, tissue area. *p < 0.05, **p < 0.01 by two‐way ANOVA. [file CPR-58-e13790-s001.docx]

**Fig. S1. SCU alleviated diabetes-induced bone mass loss and senescence of cells**

**(**A) Representative micro-CT images of cortical bone. (B) Cortical bone area (Ct.ar) and cortical bone thickness (Ct. Th) (C), and cortical bone area/tissue area (Ct.ar/Tt.ar) (D). (E and F) The concentrations of P1NP and CTX-1 were determined in blood samples. *p< 0.05, **p< 0.01 by one-way ANOVA.

**
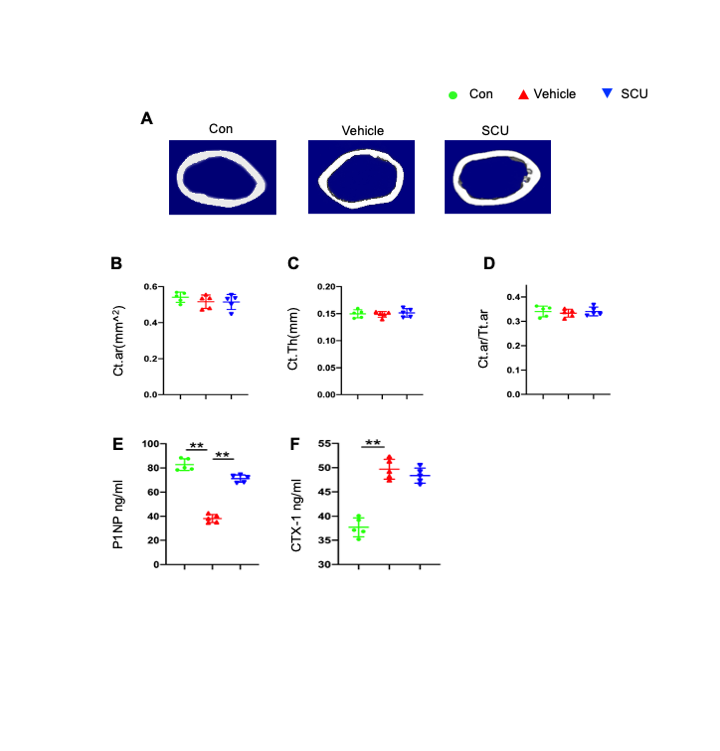
**

Fig. S2. SCU alleviated senescence and SASP in bone tissue.

Representative qRT‒PCR analyses of p21, p16, TNF-α and IL-6 mRNA expression in bone tissue from the Con, Vehicle and SCU groups (A‒D). *p< 0.05, **p< 0.01 by one-way ANOVA


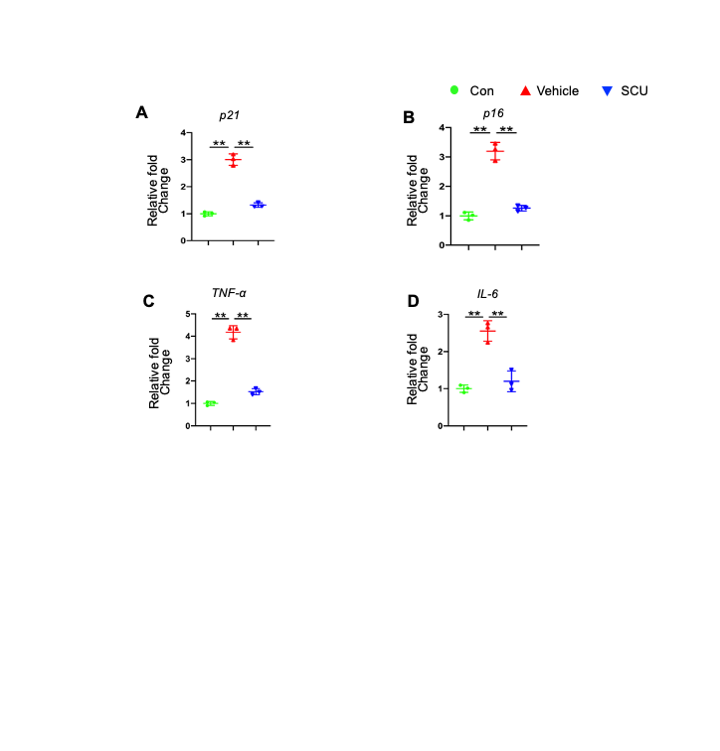


Fig. S3. Blocking *Ezh2* in LepR^+^ MSCs impaired the ability of SCU to alleviate senescence.


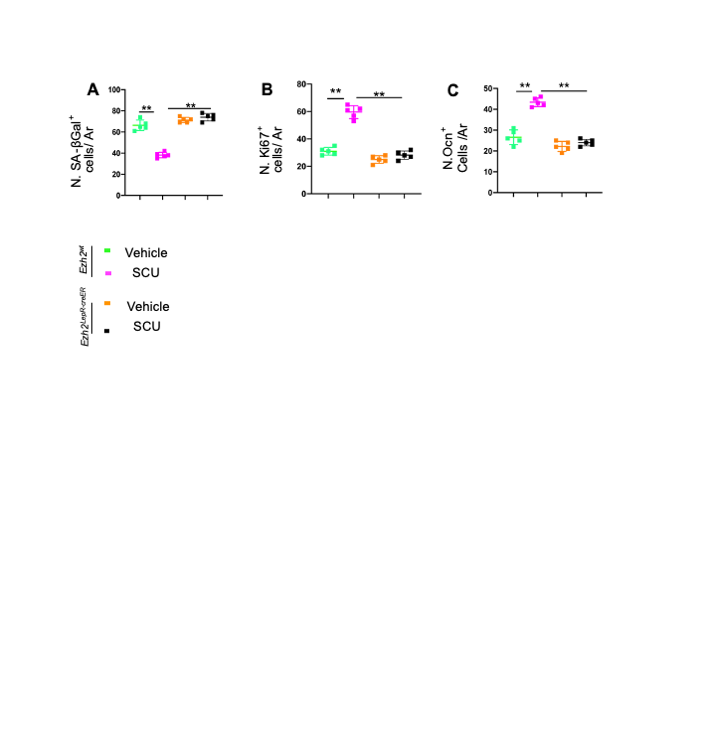


(**A**) Quantitative analysis of SA-βGal^+^ cells (blue) in primary trabecular tissue per mm^2^ tissue area (N. SA-βGal^+^ cells/Ar). (**B**) Quantitative analysis of Ki67^+^ cells (green) in primary trabeculae per mm^2^ tissue area (N. Ki67^+^ cells/Ar). **(C)** Quantitative analysis of Ocn^+^ cells in primary trabeculae per mm^2^ tissue area (N.Ocn^+^ cells/Ar). Ar, tissue area. *p< 0.05, **p< 0.01 by two-way ANOVA.
